# A new species of *Solanum* sect. *Acanthophora* (Solanaceae) from Argentina and Brazil

**DOI:** 10.3897/phytokeys.18.3903

**Published:** 2012-11-01

**Authors:** Franco E. Chiarini, Lilian Auler Mentz

**Affiliations:** 1 Instituto Multidisciplinario de Biología Vegetal, CONICET-UNC. Vélez Sarsfield 299, CC 495, Córdoba 5000, ARGENTINA; 2 Departamento de Botânica,UFRGS, Av. Bento Gonçalves, 9500, Prédio 43433, Campus do Vale, CEP 91501-970 - Porto Alegre, Rio Grande do Sul, BRAZIL

**Keywords:** Acanthophora, Argentina, Atlantic Forest, Brazil, Leptostemonum, “spiny solanum”

## Abstract

A new species of *Solanum* belonging to section *Acanthophora* (subgenus *Leptostemonum*) from Argentina and Brazil is described. *Solanum neei* Chiarini & L.A.Mentz, **sp. nov.** is found in clearings of semideciduous forests and in secondary formations, from the states of Paraná, Rio Grande do Sul and Santa Catarina in Brazil to the Misiones province in Argentina. It is morphologically similar to *Solanum incarceratum* Ruiz & Pav. from Peru, Bolivia and Western-Central Brazil, differing mainly by its pedunculate inflorescences. A key to related species is provided, as well a photograph of the holotype, a distribution map and illustration.

## Introduction

The Solanaceae is a cosmopolitan family of considerable economic importance with its centre of diversification in South America (D’Arcy 1991, Hunziker 2001). It includes 92 genera and around 2300 species, *Solanum* L. being the largest genus with ca. 1400 species. Within this genus, a remarkable natural group is subgenus *Leptostemonum* (Dunal) Bitter (the so-called “spiny solanums”), which includes cultivated representatives such as *Solanum melongena* L. (eggplant), *Solanum quitoense* Lam. (naranjilla or lulo) and *Solanum sessiliflorum* Dunal (cocona or cubiu). Other species, including *Solanum elaeagnifolium* Cav. (silverleaf nightshade), *Solanum sisymbriifolium* Lam. (sticky nightshade or wild tomato) and *Solanum carolinense* L. (horsenettle) are noxious weeds. Subgenus *Leptostemonum* includes section *Acanthophora* Dunal, whose members are characterized by the presence of simple hairs on the upper leaf surface (Levin et al. 2005, Nee 1991). This section is monophyletic (Levin et al. 2005) and comprises about 20 herbs or small shrubs adapted to disturbed areas and secondary open forests with its center of diversity in eastern Brazil. Some of its species are considered invasive weeds [e.g. *Solanum viarum* Dunal (‘‘tropical soda apple’’), naturalized in USA, Africa, and Asia (Bryson and Byrd 1994, Welman 2003), and *Solanum palinacanthum* Dunal (‘‘pocote’’, ‘‘joá bagudo’’) which invades roadsides and crop fields in Argentina and Brazil (Mentz and Oliveira 2004, Nee 1991)]. Conversely, other species of sect. *Acanthophora* are cultivated as ornamentals (e.g. *Solanum mammosum* L., ‘‘apple of Sodom’’, ‘‘cow’s udder’’ or ‘‘nipple fruit’’; and *Solanum capsicoides* All., ‘‘cockroach berry’’). Section *Acanthophora* is especially diverse with respect to the fruit, which can be a berry or a carcerulus (a fruit resembling the true berries, but with an aerial space between the seeds and the pericarp at maturity), small or relatively large (1.35–4 cm diam.), orange, red, yellow, or greenish yellow, and the seeds can be winged or not (Levin et al. 2005, Chiarini and Barboza 2009). This variability is also seen in microscopic structures, such as sclereids, layers of spongy tissue and crystals at the hypodermis, which are all related to different dispersal syndromes (Chiarini and Barboza 2009).

Nee (1979a) provided a taxonomic treatment of sect. *Acanthophora* in his doctoral thesis, that he subsequently revised slightly (Nee 1979b, 1991); in all of these he identified several probable undescribed new species known from just a few collections. Since that time, sufficient material has accumulated to formally describe one of these, and it is named in honor of Dr. Michael Nee to commemorate his work on the section.

## Taxonomic treatment

### Solanum neei

Chiarini & L.A.Mentz sp. nov.

urn:lsid:ipni.org:names:77122830-1

http://species-id.net/wiki/Solanum_neei

**Figure 1.**
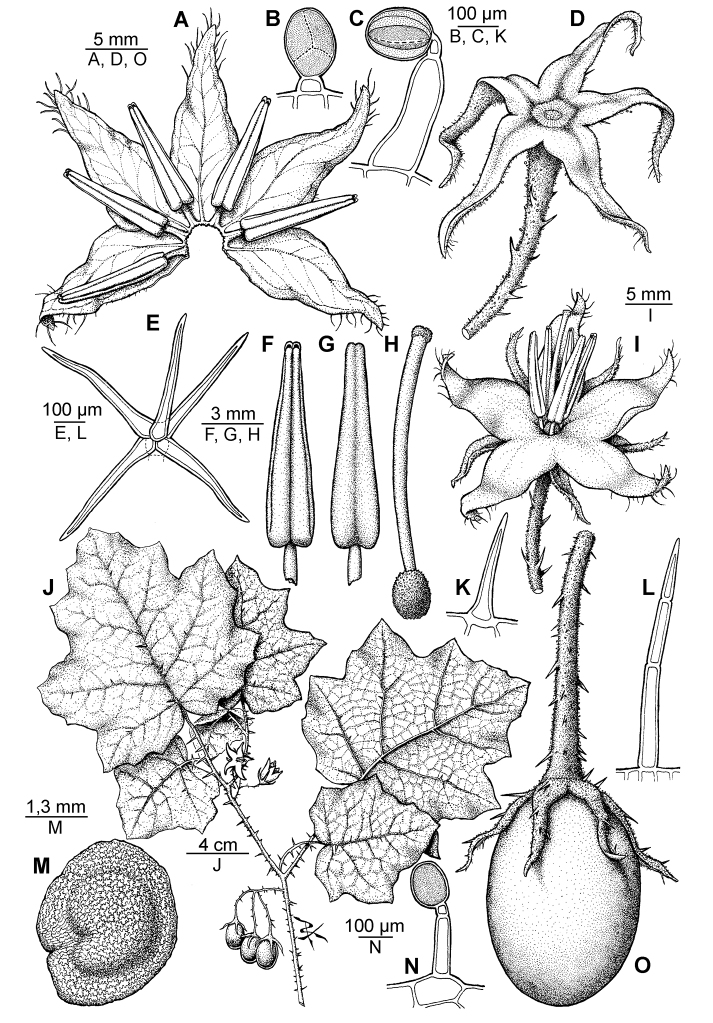
*Solanum neei*. **A** Inside view of spread open corolla **B** Glandular trichome of the ovary **C** Glandular trichome of the stem **D** Calyx **E** Stellate short-stalked trichome from the lower leaf surface **F–G** Ventral and dorsal view of the anther, respectively **H** Gynoecium **I** Flower **J** Branch with flowers and fruits **K** Unicellular trichome from the upper leaf surface **L** Multicellular uniseriate trichome from the upper leaf surface **M** Seed **N** Glandular trichome from the upper leaf surface **O** Fruit. **I** from *Tressens et al.* 6749; the rest from *Subils & Moscone 4160*. Drawn by P. Peralta.

Figs 1 [Fig F2] [Fig F3] –4

#### Diagnosis.

Differs from *Solanum incarceratum* Ruiz & Pav. by its inflorescences with unarmed or prickly peduncles 1.8–4 cm long (versus sessile inflorescences in *Solanum incarceratum*); also differs from *Solanum acerifolium* Dunal and *Solanum atropurpureum* Schrank by its unequal, linear calyx lobes (versus equal, triangular or deltoid calyx lobes in *Solanum acerifolium* and *Solanum atropurpureum*); the non-petaloid linear calyx lobes also distinguishes it from *Solanum anoacanthum* Sendtn., which has petaloid, elliptic-lanceolate calyx lobes.

#### Type.

Argentina. Misiones: Dpt. Guaraní, Predio Guaraní, 26°54'59"S, 54°12'18"W, Tramo I, borde de selva, 16-X-2002, *Solanum* *G. Tressens* et al. *6749* (holotype: CORD [CORD00006750], isotype: CTES).

#### Description.

Shrub up to 1.7 m tall. Stems sparsely pubescent with glandular hairs (stalked or sessile) and 2–5 celled simple hairs, armed with straight (sometimes recurved), broad-based prickles to 5–10 mm long, and needlelike prickles,1–2 mm long. Sympodial units di- or trifoliate. Leaves solitary or sometimes geminate, if geminate then one of the leaves about twice the size of the other; petioles 3–9 cm long, pubescent with glandular and simple trichomes and armed with acicular prickles to 1.2 cm; blades membranaceous, deltoid, 10–19 × 9–18 cm, with 2–3 pairs of shallow, broadly triangular teeth or lobes, the base truncate or cuneate to subcordate; upper surface with simple, spreading, 2–4-celled eglandular hairs and very short glandular hairs; lower surface moderately to sparsely pubescent with stellate, sessile or short-stalked trichomes, with (3) 4 (6) lateral rays and a central ray (midpoint) the same size or a little longer, and with glandular hairs and prickles on the midvein, the prickles 1–3 mm long, smaller than those of the petioles. Inflorescence extra-axillary, pedunculate, unbranched, scorpioid, (3) 5–25-flowered, with prickles to 2 mm long; peduncles up to 1.8–4 cm (2.5–5 cm in fruit) unarmed to prickly, with simple and glandular hairs; pedicels up to 3 cm, enlarged towards the apex, unarmed to prickly, with simple and glandular hairs. Flowers all perfect; calyx lobes unequal, elongate, linear-lanceolate to linear, equal to or somewhat shorter than the corolla lobes, 0.6–1.6 cm long and up to 1 mm wide (1.3–2.6 cm long in fruit), pubescent with glandular and simple hairs, sometimes also with some small prickles; corolla stellate, 2.2–2.5 cm in diameter, white, with simple hairs outside, inner surface glabrous, the lobes planar, lanceolate, 12–14 mm long × 4.5–6 mm wide; anthers attenuate, yellow, 8–9 mm long, opening by apical pores; ovary globose covered by small glandular trichomes; style whitish, 10–11 mm; stigma capitate or clavate, green. Fruits globose-ellipsoid, ca. 1.5 cm long × 1 cm wide, variegated when immature, yellow when ripe, subtended by the calyx lobes that are usually longer than the fruit (sometimes the same length or a little shorter). Seeds orbicular, compressed, winged, ± 3 mm diam. Chromosome number 2n = 24 (Acosta et al. 2005, sub nom. *Solanum* sp. 2).

**Figure 2. F2:**
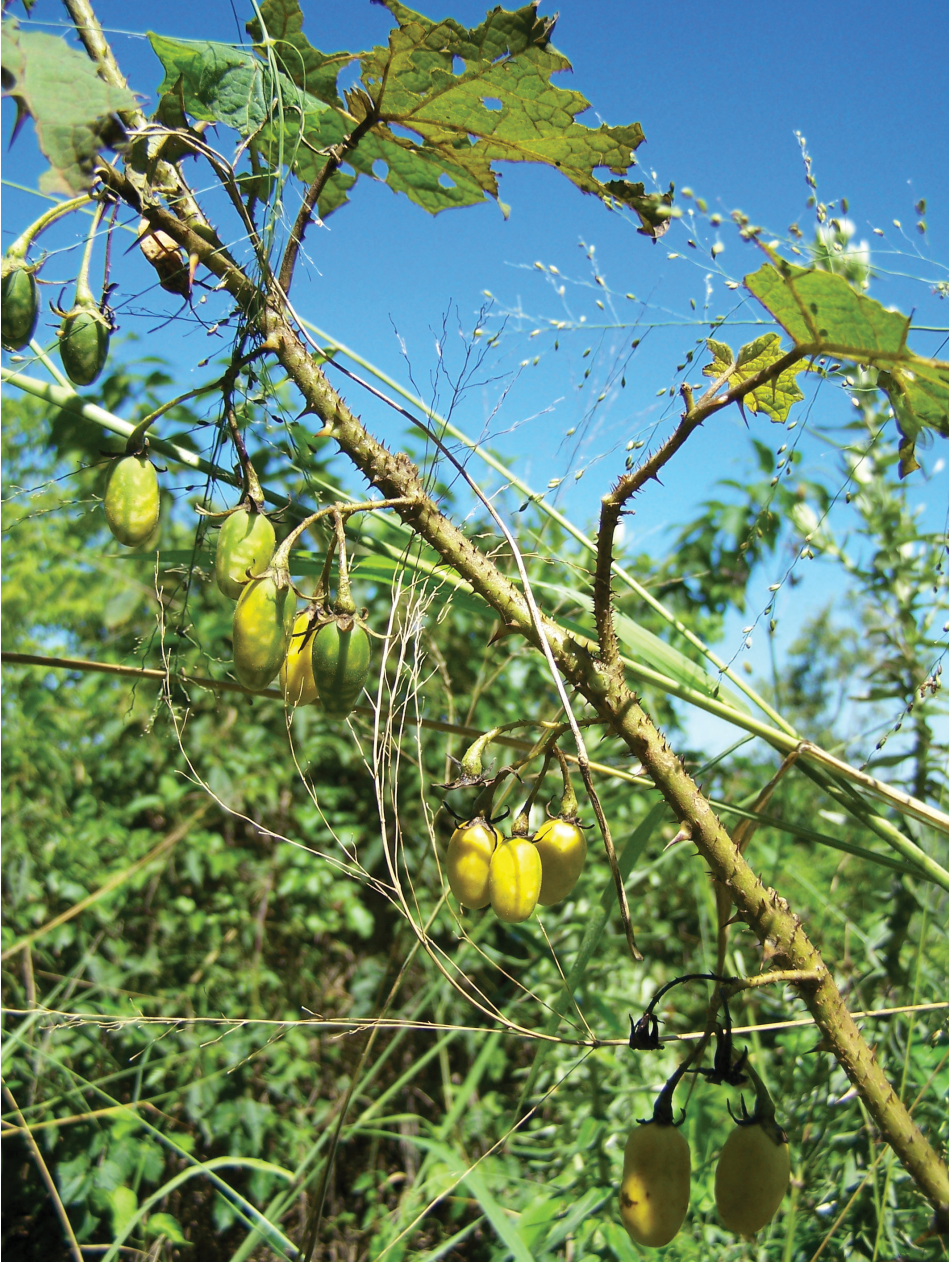
Fruiting branch of *Solanum neei* in Misiones, Argentina (*Keller 3922*, CTES).

**Figure 3. F3:**
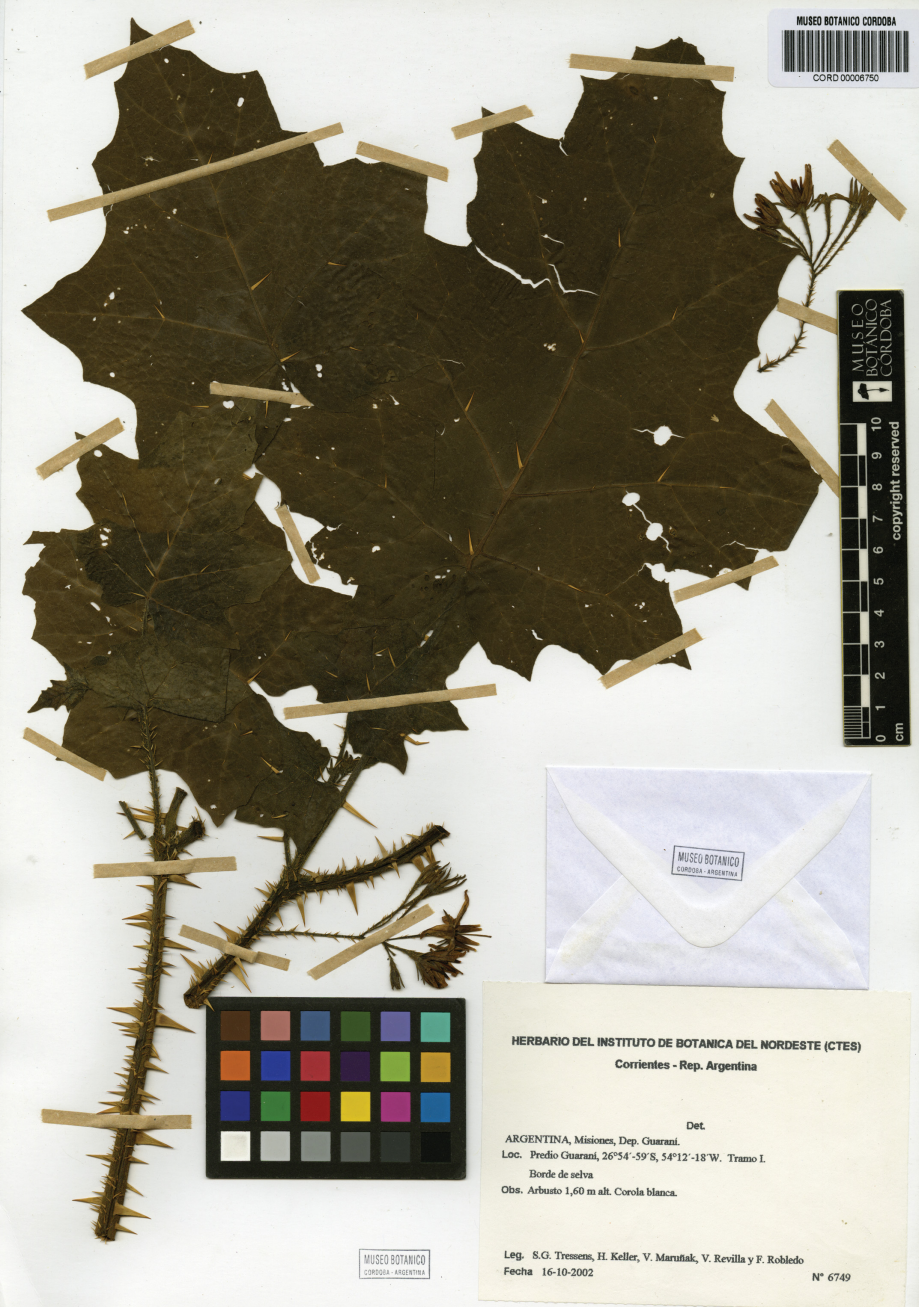
Photograph of the holotype of *Solanum neei* (*Tressens et al. 6749*, CORD 00006750).

#### Distribution

**Figure 4.**
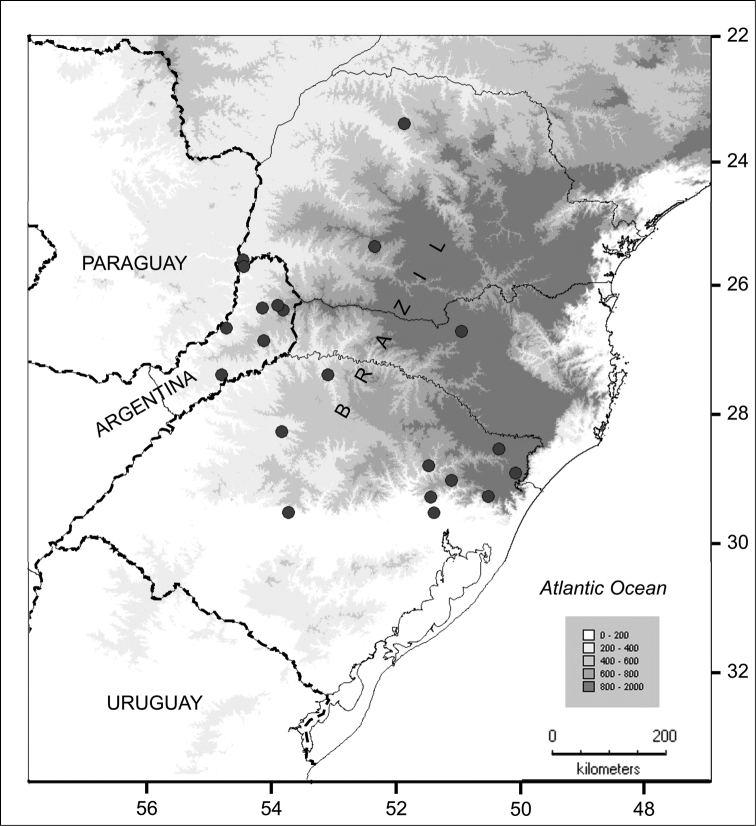
Distribution of *Solanum neei* in Argentina and Brazil.

(Fig. 4). Southern Brazil in the states of Paraná, Rio Grande do Sul and Santa Catarina, Brazil, and in Prov. Misiones, Argentina; 100–1000 m elevation. *Solanum neei* inhabits the Atlantic Forest region (Alto Paraná Atlantic forest, *Araucaria* humid forest and Serra do Mar coastal forest), in areas with 1200–2000 mm of annual precipitation.

#### Ecology.

The new species is found in clearings of semideciduous primary forests and in secondary forest.Flowering specimens have been collected from September to April; fruiting specimens from October to April.

#### Etymology.

The epithet honors Dr. Michael Nee, a specialist in Solanaceae at the Institute of Systematic Botany, New York Botanical Garden (USA). Nee’s contributions to the knowledge of this family (and particularly of genus *Solanum*) are many and remarkable, and include his Ph.D. thesis on the taxonomy of section *Acanthophora*.

#### Preliminary conservation status.

*Solanum neei* is a species of open and somewhat disturbed habitats (as are many ‘spiny solanum’ species) and seems to be fairly evenly distributed within its area (Fig. 4). The species has been regularly collected to date. Although not usually common where it occurs, it is not a species of immediate conservation concern. Applying the criteria of the IUCN (http://www.iucn.org/ ) suggests its conservation status should be ‘Least Concern’ (LC).

#### Specimens examined. 

**ARGENTINA. Misiones:** Dpt. San Javier, Cnia. Acaraguá, 29-IX-1946, *Bertoni 3032* (LIL); Dpt. Gral. San Martín, El Alcázar, propiedad Alto Paraná S.A., 23-IX-2000, *Keller 315* (CTES); Dpt. San Pedro, camino de tierra que une la RP 17 con la RN 14, a la altura de Piñalito Sur, 13-I-2007, *Keller 3922* (CTES); Dpt. Eldorado, ruta de Eldorado a B. de Irigoyen, a ± 45 km de Eldorado, 17-XI-1980, *Legname* et al. *7525* (CTES, LIL); Dpt. Iguazú, urwald picada bei den Yguazu fällen, 13-IX-1915, *Osten & Rojas 8267* (G); Dpt. San Pedro, Ruta prov. 17, 80 km al E de Eldorado, 22-I-1973, *Schinini & Fernández 5978* (SI); Dpt. Iguazú, Ruta 12, rumbo a Bosetti, a ± 5 km del límite del PN, 29-V-1987, *Subils & Moscone 4160* (CORD); Dpt. Guaraní, Predio Guaraní, 26°54'59"S, 54°12'18"W, sendero CiFOR, 27-IV-1999, *Tressens* et al. *6106* (CTES).

**BRAZIL. Paraná:** Larangeiras do Sul, Rio Iguaçú, Salto Osório, 18-IV-1970, *Hatschbach 24155* (MBM); Maringá, Horto Florestal, 29-I-1970, *Hatschbach 26187* (MBM). **Rio Grande do Sul:** Rodeio Bonito, rodovia para Planalto, 6-XII-1986, *Bassan & Benedetti 746* (HAS); São Francisco de Paula, Faz. Englert, I-1944, *Buck s.n.* (PACA 11621); Caxias do Sul, Morro do Biondi, 19-XI-1951, *Cordeiro s.n.* (ICN 896); Santo Ângelo, Granja Piratini, 24-XI-1973, *Hagelund 7473* (ICN) - XII-1941, *Leite 743* (NY) - São Salvador, 12-I-1941, *Leite 2189* (SP); Ijuí, Augusto Pestana, 27-IX-1953, *Pivetta* 946 (PACA); São Francisco de Paula, Potreiro Novo, Tainhas, 23-II-1978, *Sehnem 15906* (PACA 72218); Veranópolis, 4° seção, 3-IV-1983, *Silveira 633* (HAS); Montenegro, Kappesberg, 16-XII-1935, *Rambo 2198* (PACA); Caxias do Sul (São Francisco de Paula) Vila Oliva, 6-I-1946, *Rambo 31113* (B, PACA, LIL 199948); Bom Jesus, Fazenda da Ronda perto Vacaria, 30-XII-1946, *Rambo 34640* (PACA); Cambará do Sul, perto S. Francisco de Paula, II-1948, *Rambo 36080* (PACA). **Santa Catarina:** Caçador, Rio do Bugre, 7-XII-1962, *Klein 3449* (HBR).

#### Discussion.

This species is undoubtedly placed within sect. *Acanthophora* due to the lack of stellate hairs and the presence of simple hairs on the upper leaf surfaces. Because of its flattened and winged seeds, *Solanum neei* would be placed in the Pterosperma group of sect. *Acanthophora* in the taxonomic scheme of Nee (1979a; see Levin et al. 2005 for an explanation of Nee’s subsectional groups). However, Levin et al. (2005) demonstrated that the groupings based on seed morphology are unnatural, although they did not sample all species of the section. Therefore, the phylogenetic position of *Solanum neei* within sect. *Acanthophora* is unknown.

Morphologically, *Solanum neei* resembles *Solanum incarceratum*, but the well developed peduncles of the inflorescences (Figs 1, 2) distinguish it from the latter, which has sessile or almost sessile inflorescences. Other characteristics that also help to distinguish these two species are the leaf shape (barely lobed in *Solanum incarceratum*), the fruit shape (globose in *Solanum incarceratum*) and the distribution range: *Solanum incarceratum* inhabits Peru, Bolivia and the states of Bahia, Goiás and Minas Gerais (Brazil), therefore it would overlap with *Solanum nee* only in a small area in the Brazilian state of Paraná. *Solanum anoacanthum* has also some resemblance to *Solanum neei*: both species have long calyx lobes that are equal to or longer than the corolla lobes and longer than half of the fruit, but in *Solanum anoacanthum* the lobes are wider and petaloid, whilst in the latter, they are linear-lanceolate to linear and not more than 1 mm wide.

Some species of sect. *Acanthophora* have derived breeding systems and are andromonoecious (with both long- and short-styled flowers in the same inflorescence, e.g., *Solanum capsicoides*), but the new species seems not to be andromonoecious, since all specimens studied had inflorescences with only long-styled flowers.

A specimen belonging to *Solanum neei* was placed by Nee (1991) under the name *Solanum* species B, and later Mentz and Oliveira (2004) described and illustrated it under‘*Solanum* sp 1’, but neither of these works validly publishes it as a new species.

Key to the species of section *Acanthophora* with flattened or winged seeds in Southern Brazil/Northern-Eastern Argentina (for all members of the section in the region see Mentz and Oliveira 2004)

**Table:** 

1	Plants not andromonoecious, with all the flowers hermaphroditic; fruits smaller than 2 cm in diameter	2
–	Plants andromonoecious; fruits larger than 2 cm in diameter	5
2	Calyx lobes equal to or longer than the corolla lobes and longer than half of the fruit, elliptic-lanceolate to linear	3
–	Calyx lobes markedly shorter that the corolla lobes and shorter than the half of the fruit, deltoid to narrowly triangular	6
3	Calyx lobes not petaloid, linear-lanceolate to linear, up to 1 mm wide and 6-16 mm long	4
–	Calyx lobes petaloid, elliptic-lanceolate, ca. 2–4 mm wide and 14–20 mm long	*Solanum anoacanthum* Sendtn.
4	Penduncle of inflorescences very short or absent	*Solanum incarceratum*Ruiz & Pav.
–	Penduncle of inflorescences well developed, up to 1.8–4 cm long	*Solanum neei* Chiarini & L.A.Mentz
5	Fruits yellow or greyish-green. Stems sprawling, pubescent with 1-3-celled simple eglandular hairs and some glandular hairs and armed with profuse acicular prickles	*Solanum platense* Dieckmann
–	Fruits orange-red. Stems erect to prostrate, glabrous or pubescent with a few simple 4-6-celled eglandular hairs and armed with some acicular or recurved flattened prickles	*Solanum capsicoides* All.
6	Leaves entire or with 3–5 triangular entire lobes	*Solanum acerifolium* Dunal
–	Leaves clearly lobed or pinnatifid, the lobes sinuate or again lobed	7
7	Upper leaf surfaces almost glabrous at maturity, with only some stellate hairs on the midvein; blades deeply lobed. Stem prickles reflexed. Calyx and corolla glabrous or with a few hairs. Berries orange, globose, 15–20 mm in diameter	*Solanum atropurpureum*Schrank
–	Upper leaf surfaces with evident and persistent simple hairs; blades sinuate to lobed. Stem prickles straight. Calyx with bristle-like hairs up to 3 mm long; corolla hispid. Berries yellowish, ovoid, 11–15 mm in diameter	*Solanum tenuispinum* Rusby

## Supplementary Material

XML Treatment for Solanum neei
